# Postoperative Complications in Patients With the Preoperative COVID-19 Infection at King Fahad Specialist Hospital: A Retrospective Cohort Study

**DOI:** 10.7759/cureus.50037

**Published:** 2023-12-06

**Authors:** Mohammed Yehya Momeeh, Meshal M Alrumayh, Khiloud Ahmed, Fahad L Alharbi

**Affiliations:** 1 Anesthesia, King Fahad Specialist Hospital, Buraidah, SAU; 2 Anesthesia, Maternity and Children Hospital, Buraidah, SAU

**Keywords:** surgery during covid-19, post-operative complication, anesthesiology, surgery, covid-19

## Abstract

Background: Coronavirus disease (COVID-19) is an infectious disease caused by a new coronavirus strain. Indeed, the timing of COVID-19 infection before surgery plays an important role in the surgery outcomes and complications.

Objective: In this study, we aimed to assess the prevalence and predictors of postoperative complications for patients who underwent surgery with previous COVID-19 infection.

Methodology: This was a retrospective hospital-based study which was conducted on 75 patients who had been infected with COVID-19 and underwent surgery.

Results: We included 75 patients. The time between COVID-19 infection and the surgery was from one to six months in 52% of patients, 24% of patients were more than six months, and 24% of patients were less than one month. In this study, most of the patients had no complications (77.3%) while 22.7% of patients had complications that were mainly respiratory (n= 13). The overall mortality in our study was 5.3%. There was a significant association between comorbidity and postoperative complications, and the status of COVID-19 preoperative and postoperative complications (p < 0.01) but not patients’ age as well as the type of anesthesia and postoperative complication (p > 0.05).

Conclusion: Respiratory complications were the most common postoperative complications in patients who had surgery after COVID-19 infection. Comorbidity and COVID-19 status were significantly associated with higher postoperative complications. Mortality was relatively small. We recommend extreme care to patients with COVID-19 infection prior to surgery to decrease the COVID-19 hazards that develop post-surgery.

## Introduction

Coronavirus disease (COVID-19) is an infectious disease caused by a new coronavirus strain. COVID-19 causes mild to moderate respiratory disease which many patients recover without requiring special treatment. On the other hand, older patients and patients with comorbidities (like diabetes, cardiovascular disease, chronic respiratory disease, and cancer) are more likely to suffer from serious types of disease [1]. To provide anesthetic and surgical care to COVID-19 patients, healthcare workers have to reorganize surgical platforms, personal protective equipment protocols, and in-hospital patient trajectories to prevent a viral spread to healthcare workers and other patients in addition to decreasing the COVID-19 hazards [2-4]. During the COVID-19 pandemic surge, many elective procedures were postponed and only emergency ones were performed in order to get the maximum beneficial outcomes for the patients and decrease the workload on the overwhelmed hospitals with many COVID-19 patients [5]. However, after the COVID-19 cases started to decrease together with the introduction of vaccination, elective procedures were done for all patients considering the COVID-19 precautions before surgery to protect them and the hospital care workers from the negative COVID-19 outcomes [6]. It is worth mentioning that COVID-19 patients had significantly higher postoperative complications than their peers without COVID-19 infection [7]. Indeed, the timing of COVID-19 infection before surgery plays an important role in the surgery outcomes and complications. In a US population-based study, patients who contracted COVID-19 infection 0-8 weeks before surgery were significantly associated with postoperative complications rather than those who were allocated to surgery after at least eight weeks of COVID-19 infection [8]. In this study, we aimed to assess the prevalence and predictors of postoperative complications for patients who underwent surgery with previous COVID-19 infection.

## Materials and methods

This study was a retrospective hospital-based study conducted at King Fahad Specialist Hospital, which has a well-equipped emergency department and operating theatre including all the sub-specialties and also includes a COVID-19 department and intensive care unit (ICU).

Inclusion criteria

We included patients with previous COVID-19 who had surgery after the infection and aged from 19 to 80 years old.

Exclusion criteria

We excluded patients with incomplete data, paediatrics patients and those who refused to participate in this study.

Sample size

We aimed to recruit 60-80 patients from both genders, aged from 19 to 80 that were scheduled for elective and emergency general surgery, and obstetrics operations after contracting COVID-19 infection. The sampling technique was done using www.randomizer.org and a software application program called Epi Info™ (Centers for Disease Control and Prevention, USA) for calculations. We used simple random sampling to obtain our data.

After obtaining approval from the King Fahad Specialist Hospital and the National Committee of Bioethics, we included 75 patients with a confirmed preoperative COVID-19 infection who underwent surgery between October 2020 and August 2021. Data for this study was collected from the statistical and public health departments. The data collector obtained patients' phone numbers from the hospital system and contacted them or their families to conduct a survey. The survey included personal data, past medical history, details about COVID-19 infection symptoms, duration and complications preoperatively, how long the recovery took, the type of surgery, urgency of operation, the presence of signs, symptoms, complications developed after the operation, which type of complication, the course of treatment and outcome.

Data entry and analysis

Data was entered using Microsoft Excel (Microsoft® Corp., Redmond, WA) to be cleaned and coded, and then Statistical Package for the Social Sciences (IBM SPSS Statistics for Windows, IBM Corp., Version 26.0, Armonk, NY) was used to analyze the data. Data was reported in the form of frequency tables. To investigate the association between postoperative complications and other patient’s data, we ran a Chi-Square test. A P-value less than .05 was considered significant.

## Results

In this study, two-fifths of our patients had an age of >61 years old, 38.7% of our patients aged 19-40 years old and 21.3% had an age of 41-60 years old (Table 1).

**Table 1:** Characteristics of participants DM: diabetes mellitus, HTN: hypertension, SLE: systemic lupus erythematosus, HDU: high dependency unit, ICU= intensive care unit

Variable		Frequency	Percent
Age	19-40 years	29	38.7
	41-60 years	16	21.3
	> 61 years	30	40.0
Sex	Female	48	64
Comorbidity	None	28	37.3
	Multiple comorbidities	15	20.2
	DM	13	17.3
	HTN	11	14.7
	Asthma	4	5.3
	Renal disease	1	1.3
	SLE	1	1.3
	Smoker	2	2.6
COVID-19 status	Home isolation with mild symptoms	45	60
	Home isolation with oxygen	16	21.3
	HDU	7	9.3
	ICU	4	5.3
	Asymptomatic	3	4
Time from COVID-19 to surgery	<1 month	18	24
	1-6 months	39	52
	>6 months	18	24
Type of surgery	Minor surgery	33	44
	General surgery	25	33.3
	Orthopedic	9	12.0
	Obstetrics and gynecological operation	8	10.7
Surgical emergency	Elective	50	66.7
	Emergency	25	33.3
Type of anesthesia	General anesthesia	48	64.0
	local infiltration	11	14.7
	Regional	16	21.3

Most of the participants were females with a prevalence of 64%; while the males’ prevalence was 36%. One-fifth of our patients had no comorbidities; while the prevalence of diabetes mellitus, hypertension, asthma, renal disease, systemic lupus erythematosus and current smoker were 17.3%, 14.7%, 5.3%, 1.3%, 1.3% and 2.6%, in order. Regarding the COVID-19 status preoperatively, most of the patients had home isolation with mild symptoms 45 (60%), one-fifth needed home isolation with oxygen, seven (9.3%) and four (5.3%) patients needed a high dependency unit (HDU) and ICU admissions. Only three patients were asymptomatic. Regarding the time between COVID-19 infection and the surgery, 52% of patients had COVID-19 one to six months before surgery, 24% and 24% of patients were infected > six months and < one month before surgery, in order. In this study, 44% of surgeries were minor surgery, followed by general surgery at 33.3%, orthopaedic at 12% and obstetrics and gynaecological operation at 10.7%. In addition, most of the operations were elective 66.7% while the rest were emergency operations. Regarding the type of anaesthesia, general anaesthesia was the most frequent (64%), followed by regional (21.3%) and local infiltration (14.7%).

In this study, most of the patients had no complications (77.3%) while 22.7% of patients experienced complications. Most of the complications were respiratory (n= 13) (Table 2).

**Table 2:** Postoperative complications ARDS: acute respiratory distress syndrome, DVT: deep venous thrombosis

Variable		Frequency	Percent
Postoperative complication	No	58	77.3
	Yes	17	22.7
Respiratory complications (n= 13)	ARDS	6	46.1
	Acute lung injury	2	15.4
	Pneumonia	2	15.4
	Pulmonary embolism	2	15.4
	Postoperative hypoxia	1	7.6
Non-respiratory complications	DVT	3	66.7
	Sepsis	1	33.3

Among patients with respiratory complications, nine patients received mechanical ventilation and the rest received non-invasive ventilation. Regarding the outcome of treatment of the respiratory complications, ten patients recovered and three patients died. Regarding the patients who had non-respiratory complications, three had deep venous thrombosis (DVT) and only one patient had sepsis. Considering the treatment needed for non-respiratory complications, one patient was treated in the critical care unit, whereas only three patients were treated in the ward. Three patients recovered and one patient died. The overall mortality in our study was 5.3%.

There was a significant association between comorbidity (Table 3) and the status of COVID-19 (Table 4) and postoperative complications (p < 0.01). In contrast, there was no significant association between patients’ age and postoperative complications (Table 5) as well as the type of anaesthesia and postoperative complication (Table 6) (p > 0.05).

**Table 3:** Association of effects of preoperative COVID-19 and postoperative complications *Three asymptomatic patients were added to this group. HDU: high dependency unit

The effect of COVID-19 preoperatively		Postoperative complications		P value
		No	Yes	
HDU	Frequency	5	2	<0.001
	Percent	71.4%	28.6%	
Home isolation with mild symptoms*	Frequency	46	2	
	Percent	95.8%	4.4%	
ICU	Frequency	1	3	
	Percent	25.%	75%	
Home isolation with oxygen	Frequency	6	10	
	Percent	37.5%	62.5%	

**Table 4:** Association between complications and comorbidity DM: diabetes mellitus, HTN: hypertension, SLE: systemic lupus erythematosus

Comorbidity type		Postoperative complication		P value
		No	Yes	
Asthma	Frequency	0	4	0.008
	Percent	0%	100%	
DM	Frequency	10	3	
	Percent	76.9%	23.1%	
HTN	Frequency	8	3	
	Percent	72.7%	27.3%	
Multiple co-morbidities	Frequency	14	1	
	Percent	93.3%	6.7%	
No comorbidity	Frequency	23	5	
	Percent	82.1%	17.9%	
Renal disease	Frequency	1	0	
	Percent	100%	0%	
SLE	Frequency	1	0	
	Percent	100%	0%	
Smoker	Frequency	1	1	
	Percent	0%	100%	

**Table 5:** Association between age and postoperative complications

Age		Postoperative complications		P-value
		No	Yes	
19-40 years	Frequency	20	9	0.20
	Percent	69.0%	31.0%	
41-60 years	Frequency	13	3	
	Percent	81.3%	18.8%	
> 61 years	Frequency	25	5	
	Percent	83.3%	16.7%	
Total	Frequency	58	17	
	Percent	77.3%	22.7%	

**Table 6:** Association between anaesthesia type and postoperative complications

Anesthesia type		Postoperative complications		P value
		No	Yes	
General	Frequency	35	13	0.9
	Percent	72.9%	27.1%	
Local infiltrations	Frequency	11	0	
	Percent	100%	0%	
Regional	Frequency	12	4	
	Percent	75%	25%	

## Discussion

In our study, 17% of the study population (75 patients) experienced respiratory complications. This differs from what is shown by Dimitry et al. [9], who found a higher prevalence of respiratory complications that had a prevalence of 51% of the total patients. It was much higher than the percentage we found, but in reality, their study population was larger than ours with 1128 included COVID-19 patients. Furthermore, the complications they reported were related to only the emergency surgery while in our study only 33.3% were allocated to emergency surgery.

Regarding the mortality rate, a study conducted in 2021 by Richard Shaw et al. [10], on head and neck cancer surgery during the COVID-19 pandemic in 26 countries, in which 1137 patients were enrolled. The reported overall 30-day mortality was 1.2%. Twenty-nine patients (3%) tested positive for severe acute respiratory syndrome coronavirus 2 (SARS-CoV-2) within 30 days of surgery, 13 (44.8%) of these patients developed severe respiratory complications and three patients (10.3%) died [10].

We found no significant association between the type of anaesthesia and postoperative complications. This indicates that the type of anaesthesia had no impact on the postoperative complication. However, we still recommend large sample-sized studies to support our results.

Regarding comorbidity and postoperative complications, our results revealed a positive association between comorbidity and postoperative complications. It is well known that comorbidity plays a vital role in postoperative outcomes at which patients with multiple comorbidities have worse outcomes [11]. This risk was enhanced by the COVID-19 infection which shows marked hazards in patients with comorbidities [12].

Regarding the association between the course of previous COVID-19 disease treatment and postoperative complications, we found a significant association between the course of the disease and postoperative complications. ICU patients and patients who needed oxygen were more vulnerable to acquiring postoperative complications. This agrees with a cohort study that was conducted by François Martin Carrier et al. [13], in 2021 regarding the postoperative outcomes in surgical COVID-19 patients in Canada, in which 44 COVID-19 patients were included in the study. Among the 44 COVID-19 patients, 31 surgeries (71%) were urgent and 16 (36%) were major. Furthermore, in this study, pulmonary complications were frequent (25%) and 30-day mortality was high (15.9%). The latter was higher in symptomatic patients (23.1%) compared to asymptomatic ones (5.6%).

Also, a study conducted by Michael E. Kiyatkin et al. [14] in 2020 regarding the increased incidence of postoperative respiratory failure in patients with preoperative SARS-CoV-2 infection in the USA, in which 778 patients with COVID-19 were included in the study. The incidence of postoperative respiratory failure was 16% versus 7% in uninfected participants (p = 0.001). Among the infected individuals, 39% exhibited symptoms of COVID-19 and postoperative respiratory failure was more common in these patients compared to asymptomatic individuals (26% vs. 9%, p = 0.04).

Limitations

We did not involve non-COVID-19 patients in our study and their postoperative complication outcomes. Our study also had a small sample size. Furthermore, the retrospective nature of our study increased the possibility of selection bias [15].

## Conclusions

Respiratory complications were the most common postoperative complications in patients who had surgery after COVID-19 infection. Comorbidity and COVID-19 status were significantly associated with higher postoperative complications. Mortality was relatively small. We recommend extreme care to patients with COVID-19 infection prior to surgery to decrease the COVID-19 hazards that develop post-surgery.

## References

[1] Struyf T, Deeks JJ, Dinnes J (2022). Signs and symptoms to determine if a patient presenting in primary care or hospital outpatient settings has COVID-19. Cochrane Database Syst Rev.

[2] Li J, Gao R, Wu G (2020). Clinical characteristics of emergency surgery patients infected with coronavirus disease 2019 (COVID-19) pneumonia in Wuhan, China. Surgery.

[3] Velly L, Gayat E, Quintard H (2020). Guidelines: anaesthesia in the context of COVID-19 pandemic. Anaesth Crit Care Pain Med.

[4] Gulinac M, Novakov IP, Antovic S, Velikova T (2021). Surgical complications in COVID-19 patients in the setting of moderate to severe disease. World J Gastrointest Surg.

[5] Moletta L, Pierobon ES, Capovilla G, Costantini M, Salvador R, Merigliano S, Valmasoni M (2020). International guidelines and recommendations for surgery during Covid-19 pandemic: a systematic review. Int J Surg.

[6] Shivkumar S, Mehta V, Vaddamanu SK (2023). Surgical protocols before and after COVID-19 - a narrative review. Vaccines (Basel).

[7] Prasad NK, Mayorga-Carlin M, Sahoo S (2023). Mid-term surgery outcomes in patients with COVID-19: results from a nationwide analysis. Ann Surg.

[8] Deng JZ, Chan JS, Potter AL (2022). The risk of postoperative complications after major elective surgery in active or resolved COVID-19 in the United States. Ann Surg.

[9] COVIDSurg Collaborative (2020). Mortality and pulmonary complications in patients undergoing surgery with perioperative SARS-CoV-2 infection: an international cohort study. Lancet.

[10] COVIDSurg Collaborative (2021). Head and neck cancer surgery during the COVID-19 pandemic: an international, multicenter, observational cohort study. Cancer.

[11] Kim W, Song KY, Lee HJ, Han SU, Hyung WJ, Cho GS (2008). The impact of comorbidity on surgical outcomes in laparoscopy-assisted distal gastrectomy: a retrospective analysis of multicenter results. Ann Surg.

[12] Biswas M, Rahaman S, Biswas TK, Haque Z, Ibrahim B (2020). Association of sex, age, and comorbidities with mortality in COVID-19 patients: a systematic review and meta-analysis. Intervirology.

[13] Carrier FM, Amzallag É, Lecluyse V (2021). Postoperative outcomes in surgical COVID-19 patients: a multicenter cohort study. BMC Anesthesiol.

[14] Kiyatkin ME, Levine SP, Kimura A (2021). Increased incidence of post-operative respiratory failure in patients with pre-operative SARS-CoV-2 infection. J Clin Anesth.

[15] Mulargia F (2001). Retrospective selection bias (or the benefit of hindsight). Geophys J Int.

